# The Pocket Pharmacy with Therapeutic Index

**Published:** 1892-10

**Authors:** 


THE POCKET PHARMACY WITH THERAPEUTIC INDEX. A resumé of the clinical applications of remedies adapted to the pocket-case, for the treatment of emergencies and acute diseases. By John Aulde, M. D., of Philadelphia. Pages 204. Price—. New York: D. Appleton & Co. 1892.

The first twelve pages of this volume are taken up with the *Preface and Introduction*, in which a masterly effort is made to justify its appearance, along with so many other better and less fragmentary works. As one reads along through the preface, he sees between the lines, at a distance, that there is an enterprising manufacturing pharmacist at the bottom of the work, and sure enough, at the last sentence the author expresses his obligation to a well-known Philadelphia firm for "valuable suggestions." Dr. Aulde begins the business part of the book with "Acetanilide Compound," then with heavy faced type he recounts forty-nine different diseases in which the remedy is indicated. The writer thought he would send for the "compound" and stop everything else. B.

